# Motor and respiratory intensive rehabilitation in bedridden patients

**DOI:** 10.1186/cc11134

**Published:** 2012-03-20

**Authors:** E Canedo, V Nunes Velloso, L Calejman, N Leidi

**Affiliations:** 1 Hospital de Infecciosas F.J. Muñiz, Buenos Aires, Argentina

## Introduction

Inability to play significant social roles due to a pattern of motor disability affects the quality of a person's life, and is where the motor and respiratory rehabilitation process takes fundamental importance. This disability prevents one to function independently in basic tasks such as dressing and feeding and in more complex tasks such as handling in public and/or work. It can also be a constraint for the dependent patient in personal care activities. The objective of a motor rehabilitation plan is to reduce the impact caused by this alteration of motor ability, facilitating the restoration of functional patient capacity so they can effectively engage in occupations, reaching the highest level of functional independence possible.

## Methods

A cross-sectional retrospective descriptive and observational study of rehabilitation of bedridden patients in hospital from January 2010 to June 2011. The programme is implemented in Section 30 (21, 9, and 20 rooms). The inclusion criteria for the rehabilitation programme were patients of both sexes, without age limit, inpatient of Hospital F.J. Muñiz coming from the ICU, in bedridden condition (limitation or motor disability in which the patient cannot move or perform activities of daily living and must depend on the care of others), with Barthel scale value 0 to 35 with total or severe dependence and stability hemodynamics.

## Results

We included patients who met the inclusion criteria. The program presented an intensive character in terms of the frequency of weekly sessions as the number of exercises implemented in the form was specified according to the pathology of the patient. Ninety percent of patients were male. The median age was 41 years. The predominant infectious pathology was pulmonary tuberculosis (90%), cerebral toxoplasmosis (50%), spastic paraplegia (6%), bilateral pneumonia (6%), and fumigares aspergillosis (6%). The profit was 100% of kinesic treatment adherence, 94% of cases won full independence valued on the Barthel scale with a value of 100, and a single case achieved independence moderated by the presence of spastic paraplegia.

## Conclusion

The intensive rehabilitation programme presented a great benefit for hospitalized patients; taking them from being bedridden to total independence in the AVD, the outpatient had better social and labor conditions.

